# Cost-Effectiveness of Exercise Therapy in Patients with Intermittent Claudication—A Comparison of Supervised Exercise, Home-Based Structured Exercise, and Walk Advice from the SUNFIT Trial

**DOI:** 10.3390/jcm12165277

**Published:** 2023-08-14

**Authors:** Hildigunnur Ulfsdottir, Maria Bäck, Åsa Cider, Lennart Jivegård, Anna Sandberg, Joakim Nordanstig, Mikael Svensson

**Affiliations:** 1 School of Public Health and Community Medicine, Institute of Medicine, Sahlgrenska Academy, University of Gothenburg, 405 30 Gothenburg, Sweden; hildigunnur@sidekickhealth.com; 2 Sidekick Health, 413 90 Gothenburg, Sweden; 3 Department of Occupational Therapy and Physiotherapy, Sahlgrenska University Hospital, 416 85 Gothenburg, Sweden; maria.m.back@vgregion.se (M.B.); asa.cider@neuro.gu.se (Å.C.); anna.m.sandberg@vgregion.se (A.S.); 4 Department of Molecular and Clinical Medicine, Institute of Medicine, Sahlgrenska Academy, University of Gothenburg, 405 30 Gothenburg, Sweden; lennart.jivegard@vgregion.se; 5 Department of Health and Rehabilitation/Physiotherapy, Institute of Neuroscience and Physiology, Sahlgrenska Academy, University of Gothenburg, 405 30 Gothenburg, Sweden; 6 Health Technology Assessment Centre Region Västra Götaland, Sahlgrenska University Hospital, 416 85 Gothenburg, Sweden; 7 Department of Vascular Surgery, Sahlgrenska University Hospital, 416 85 Gothenburg, Sweden; 8 Department of Pharmaceutical Outcomes & Policy, College of Pharmacy, University of Florida, 1225 Center Drive, Gainesville, FL 32610, USA

**Keywords:** peripheral artery disease, intermittent claudication, exercise therapy, quality-adjusted life-years, cost-effectiveness analysis

## Abstract

Hospital-based supervised exercise (SEP) is a guideline-recommended intervention for patients with intermittent claudication (IC). However, due to the limited availability of SEP, home-based structured exercise programs (HSEP) have become increasingly popular alongside the “go home and walk” advice. We evaluated the cost-effectiveness of walk advice (WA) with Nordic pole walking vs. SEP combined with WA or HSEP combined with WA. We used data from the SUNFIT RCT (NCT02341716) to measure quality-adjusted life-years (QALYs) over a 12-month follow-up, and economic costs were obtained from a hospital cost-per-patient accounting system. Incremental cost-effectiveness ratios (ICERs) were calculated, and uncertainty was assessed using nonparametric bootstrapping. The average health-care-cost per patient was similar in the WA (EUR 1781, n = 51) and HSEP (EUR 1820, n = 48) groups but higher in the SEP group (EUR 4619, n = 50, *p*-value < 0.01). Mean QALYs per patient during the follow-up were similar with no statistically significant differences. The findings do not support SEP as a cost-effective treatment for IC, as it incurred significantly higher costs without providing additional health improvements over WA with or without HSEP during the one-year observation period. The analysis also suggested that HSEP may be cost-effective compared to WA, but only with a 64% probability.

## 1. Introduction

Peripheral arterial disease (PAD) is an example of a chronic noncommunicable disease that causes a large economic and social burden globally [1]. Intermittent claudication (IC) is a common symptomatic presentation of PAD and an important symptomatic manifestation of systemic atherosclerosis, with significant cardiovascular morbidity and mortality [2]. It is estimated that IC affects around 20–40 million persons worldwide, with an increasing prevalence mainly due to aging populations [3]. In Sweden, the prevalence is estimated at approx. 7% in individuals ≥60 years of age [4]. IC symptoms are caused by stenosis or obstruction in the lower limb arteries and are characterized by pain or discomfort in the legs brought on by walking and relieved at rest [2]. IC can impair walking ability, limit activities of daily living, decrease functional status, and negatively affect general health-related quality of life (HRQoL) [2,5,6,7]. 

The main treatment goals of management of IC are: (i) to reduce the risk of major cardiovascular events with risk factor control and secondary preventive pharmacotherapy, and (ii) to increase walking ability and HRQoL [8,9]. As exercise therapy has beneficial effects on both these treatment goals, it is considered the first-line treatment of IC [8,10]. Revascularization is another commonly used treatment option. However, evidence to support its long-term benefits and cost-effectiveness is limited [11,12].

The literature generally refers to the following exercise frameworks for patients with IC: hospital-based supervised exercise programs (SEP), home-based structured exercise programs (HSEP), and unsupervised walk advice (WA) [13]. SEP occurs in a hospital or outpatient clinic under the supervision of a health-care provider. SEP for IC is performed in 30–45 min sessions, at least three times/week, for at least 12 weeks. The exercise program includes intermittent bouts of walking to moderate-to-maximum claudication, alternating with rest periods [14]. SEP is recommended in international treatment guidelines as first-line therapy for IC limb symptoms and is generally considered the most effective approach to improve treadmill walking capacity [8,15]. However, uncertainty remains regarding patient adherence to SEP and its long-term effectiveness [13,16]. Furthermore, the availability of SEP is limited in many countries as SEP is not necessarily reimbursed [17,18].

HSEP describes a heterogeneous group of self-directed, IC-specific, home-based exercise programs with guidance provided by health-care providers. These programs commonly consist of intermittent walking exercises where the structure, follow-up, and design also include an ambition for behavioral change through health coaching activities [14,19]. HSEP is generally not considered as effective as SEP [8,20], but there is evidence supporting HSEP as a good option when SEP is unavailable or considered unsuitable [19]. Some evidence also points to HSEP being superior to SEP in increasing daily activity levels [18]. Furthermore, some patients seem to prefer HSEP over SEP [21]. 

WA includes verbal and written advice from an educated health-care provider to conduct IC pain-inducing walking sessions for 30–60 min two to three times a week, usually without any further follow-up [22]. WA is generally considered part of standard care for patients with IC, although patient adherence is often low [23].

With an increasing prevalence of IC, health-care systems will see an increased demand for health-care resources allocated to IC treatments. All health-care systems face the reality of limited resources, and one approach to assess the relative value of treatments to recommend or reimburse is to assess the cost-effectiveness—a systematic comparison of both economic costs and health outcomes with alternative treatments. Cost-effectiveness evidence on SEP, HSEP, and WA remains limited and, to our knowledge, includes two studies (from the Netherlands and the UK) that suggest that SEP is a cost-effective treatment compared to WA [24,25]. However, there is a lack of evidence comparing SEP to HSEP and HSEP to WA. 

This study accordingly aimed to determine which of the interventions, SEP, HSEP, or WA, is the preferred treatment option from a cost-effectiveness perspective for patients with IC in a Swedish context.

## 2. Materials and Methods 

### 2.1. Study Design

This cost-effectiveness analysis was based on data from the SUNFIT trial (ClinicalTrials.gov identifier NCT02341716). The SUNFIT trial was a three-armed, prospective, multicenter, randomized controlled trial (RCT) that compared the one-year efficacy of six months of SEP, HSEP, and WA on walking distance, muscle endurance, and HRQoL among patients with mild to severe IC. The trial was performed at the vascular surgery departments of Sahlgrenska University Hospital, Gothenburg, the Region Hospital of Karlstad, and Södra Älvsborg Hospital, Borås, Sweden. The cost-effectiveness analysis reported in this paper was only based on patients from the Sahlgrenska University Hospital, where both cost and HRQoL data were accessible, and covers 90% of all patients in the study. The study design and clinical efficacy endpoints have been published previously [26], with the main clinical result being that no difference was detected between treatment alternatives in terms of six-minute walking test distances. 

### 2.2. Patients and Interventions

The inclusion criteria were at least six months of mild-to-severe IC of vascular origin with an ankle-brachial index (ABI) of less than 0.9 and/or a postexercise ABI reduction of 30% or more. Exclusion criteria included previous revascularization for IC in the last 3 months, if revascularization was deemed necessary within 12 months, cognitive dysfunction, inability to perform the six-minute walk test, and the inability to speak and understand Swedish. Patients that fulfilled the inclusion criteria and had no exclusion criteria [26] were offered enrolment in the trial and were randomly allocated (using adaptive stratified sampling) to one of the three intervention groups: (1) SEP + WA, (2) HSEP + WA, or (3) WA alone. Regardless of group allocation, all participating patients in the trial received free Nordic poles and were advised by a vascular surgeon to perform limb-symptom-inducing Nordic pole walking for at least 30 min three times weekly for six months. Furthermore, all patients received detailed disease-specific written information, best medical treatment, and smokers were offered assistance with smoking cessation. Patients in the SEP or HSEP groups received either an individually adjusted SEP or an HSEP. Both these exercise frameworks consisted of 50 min of aerobic walking exercises and resistance training, offered three times a week for six months [26]. Biweekly follow-up was arranged for both groups; through phone consultations by the physiotherapist in the HSEP group and as individual face-to-face meetings with the physiotherapist in the SEP group. Full details on the SEP and HSEP interventions have been described in full in a previous publication. 

### 2.3. Health Outcomes

Health outcomes were assessed in terms of quality-adjusted life-years (QALYs). QALY is a health status measure that combines the time spent in a specific health state with a corresponding self-assessed HRQoL score. The HRQoL score is measured on an index scale where one is the best possible health status, and zero equals death [27]. One QALY can be interpreted as one life-year in full health. HRQoL was assessed using the 36-item short-form health survey (SF-36) at baseline and three, six, and twelve months [28]. The SF-36 score was converted to a preference-based measure of health status, the SF-6D index score, based on the scoring method created by Brazier and colleagues [29]. We used linear interpolation of the HRQoL score between measurement points. Considering the twelve months follow-up, the highest QALY sum for a patient is equal to one (i.e., someone living the entire year in perfect health).

### 2.4. Economic Costs

Total in- and outpatient PAD-treatment-related costs were accessed from the hospital cost-per-patient accounting system for each patient from the start of the trial up to a time that covered the 12-month follow-up visit and any subsequent lower limb revascularization procedures that were scheduled at the 12-month follow-up visit. Outpatient costs included all doctor, nurse, and physiotherapist visits and phone consultations. Inpatient costs included costs associated with hospitalization, lower limb revascularization procedures, foot ulcer treatments, and medical imaging. Costs were initially measured in Swedish kronor (SEK) and then converted to Euros at SEK 1 = EUR 0.099. No discounting was performed due to the time frame of the analysis.

### 2.5. Cost-Effectiveness Analysis

The cost-effectiveness analysis was carried out from a health-care payer perspective. Thus, broader potential consequences for society, such as effects on production loss or other non-health-care consequences were not included. We calculated cost-effectiveness ratios (CERs) for all potential comparisons as the difference in mean costs divided by the difference in mean QALYs between treatments (ΔCOST/ΔQALY). The CERs were compared to a predefined willingness-to-pay (WTP) threshold of EUR 50,000 per QALY, defined as a moderate threshold level in Swedish health policy per the Swedish National Board of Health and Welfare [30]. 

### 2.6. Statistical Analysis

Analyses were carried out using the intention-to-treat (ITT) principle, and the results on mean per patient QALYs and economic costs in the three respective groups were initially compared using ANOVA. The mean differences in cost and QALYs used to calculate CERs were based on linear regression analyses with the cost/QALYs as the outcome variable and a binary dummy variable indicating treatment status as explanatory variable. In the incremental QALY regression, we also controlled for baseline HRQoL score [31]. To account for sampling uncertainty and skewed data, confidence intervals and the uncertainty were assessed using nonparametric bootstrapping [32]. The results from the bootstrap analysis for ICERs were summarized in cost-effectiveness acceptability curves (CEACs) and were based on 1000 bootstrapped re-samples of the original data. 

Cost data were accessed from the hospital accounting registers and were complete for all patients, whereas HRQoL data were missing for some patients at different follow-up measurements. Alongside a complete case analysis, we therefore carried out sensitivity analyses using multiple imputations with predictive mean matching to account for missing data [33]. The predictors used in the analysis were gender, smoking, comorbidity, disease severity status, and baseline physical activity level. All analyses were carried out using Stata v.16 [34].

## 3. Results

### 3.1. Study Population and Baseline Data

The SUNFIT trial enrolment period began in September 2014 and ended in February 2018. In total, 733 patients were screened and 362 patients were eligible for participation, of which 166 (46%) were willing to participate in the trial. The most common reason why a patient declined participation was reluctance to be randomized to the SEP group. The sample size from the Sahlgrenska University Hospital included 149 patients, representing 90% of the entire sample. The distribution of patients between intervention groups included in the cost-effectiveness analysis and their baseline demographic factors and risk profiles are shown in Table 1. 

### 3.2. Health Outcomes and Economic Costs

Table 2 shows that the average HRQoL scores at baseline and at the 12-month follow-up visit were similar across all three groups. Total QALYs were assessed using both complete case (CC) analysis and multiple imputations (MI) for the 56 patients that lacked one or more follow-up HRQoL scores. Results showed no substantial or statistically significant difference in total QALYs across the three groups during the 12-month follow-up (estimates from CC and MI: WA = 0.72, HSEP = 0.72–0.74, and SEP = 0.71–0.73). 

The mean cumulative in- and outpatient cost per patient was EUR 1781 in the WA group, EUR 1820 in the HSEP group, and EUR 4619 in the SEP group. The main cost driver was the outpatient cost, which was significantly higher in the SEP group than in the HSEP and WA groups (*p* = 0.001). The outpatient cost was higher in the SEP group primarily due to more physiotherapist visits, whereas the mean number of doctor and nurse contacts were similar between all intervention groups (Supplementary Table S1). Of the 149 patients, only 10 required PAD-related inpatient care: 1 patient in the WA group, 4 in the HSEP group, and 5 in the SEP group. 

### 3.3. Cost-Effectiveness Analysis Results

Table 3 shows the cost-effectiveness analysis for the three alternative comparisons. In a comparison between HSEP and WA, HSEP was EUR 39.6 more costly per patient than WA, with 0.01 higher QALYs. None of these differences were statistically significant and can also be considered economically and clinically nonsignificant. Using the point estimates, the ICER for HSEP vs. WA was EUR 3749 per gained QALY.

In a comparison between SEP and WA, SEP was EUR 2834 more expensive per patient (*p*-value = 0.002) and had 0.01 higher QALYs (*p*-value = 0.58). Using the point estimate, the cost-effectiveness ratio of SEP vs. WA was EUR 278,981 per QALY.

Comparing SEP to HSEP shows that the incremental cost was higher (EUR 2798), whereas QALYs were lower (−0.0001). Only the difference in cost was statistically significant. Using the point estimates, this leads to interpreting HSEP to dominate SEP with lower costs and (marginally) better health outcomes.

Altogether, the results indicate that SEP is substantially more expensive without any demonstrated gain in health outcomes. The differences in QALYs for all comparisons were similar using multiple imputations to account for missing HRQoL data (Supplementary Table S2), and thus did not lead to any different interpretations as compared to the complete case analysis.

**Figure 1:**
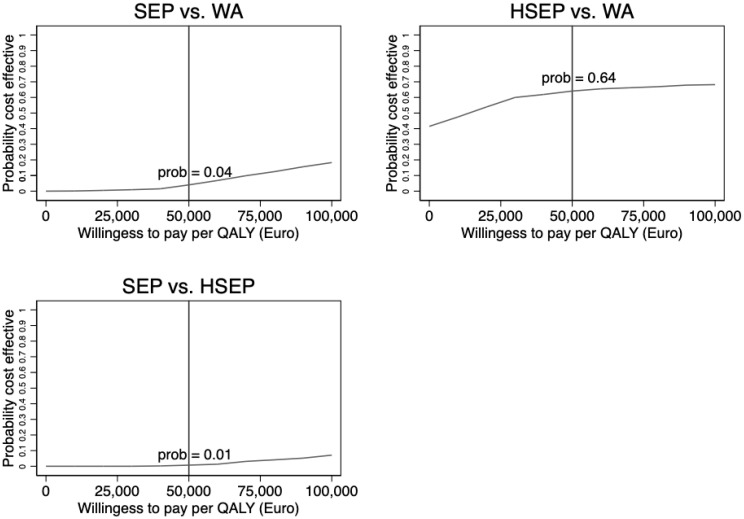
Cost-effectiveness acceptability curve for each of the three comparisons based on nonparametric bootstrapping.

Figure 1 shows the CEAC for all three comparisons based on the nonparametric bootstrapping. The CEAC shows the probability that each intervention is cost-effective (vs. the comparator) at different levels of the maximum acceptable cost per QALY. As would be expected based on the results (Table 3), the likelihood that SEP was considered cost-effective using a EUR 50,000 cost-effectiveness threshold compared to WA (4%) and HSEP (1%) was very low. 

At the same threshold level of EUR 50,000, there was a 64 percent likelihood that HSEP would be cost-effective compared with WA. 

## 4. Discussion

The results from this cost-effectiveness analysis based on the SUNFIT trial showed that SEP was more than twice as costly as HSEP and WA without demonstrating any clinically meaningful or statistically significant improvement in QALYs. The likelihood for SEP to be cost-effective compared to HSEP (or WA) was thus almost null. For the comparison between HSEP and WA, no statistically significant differences were found in costs or health outcomes. However, the point estimates indicated slightly higher costs and better health outcomes for HSEP compared to WA and an incremental cost-effectiveness ratio of about EUR 3750 per gained QALY, which is clearly below the informal Swedish willingness-to-pay threshold of EUR 50,000 per gained QALY. At the EUR 50,000 threshold, there was a 64 percent likelihood that HSEP was cost-effective compared to WA. The ICER is also well below typically used cost-effectiveness thresholds in the UK (GBP 20,000–GBP 30,000) and the US (USD 100,000–USD 150,000) [35].

Our results differ from previous research that indicated SEP as a cost-effective treatment alternative. A study from the Netherlands showed that SEP was cost-effective compared to WA based on data from an RCT where SEP was more expensive but also produced better health outcomes [25]. Another study found that SEP was cost-effective compared to unsupervised exercise, using a Markov decision model in a UK health-care context [24]. However, the authors highlighted that the cost-effectiveness of SEP was strongly linked to adherence with the exercise programs and the assumed links between exercise and reduced cardiovascular risks.

Some characteristics that differ between the previous studies and the SUNFIT trial could explain the difference in outcomes. First, the Dutch study evaluated an SEP intervention lasting up to twelve months, whereas the intervention in the SUNFIT trial lasted for six months. The difference in length of the interventions could potentially explain the lack of benefits from SEP in our study. Furthermore, adherence to the exercise program is an important factor in the likelihood of success and is often uncertain with exercise interventions [16]. Although partial or full adherence to the exercise interventions in the SUNFIT trial was 95% (HSEP) and 74% (SEP), full adherence with the interventions was indeed low (24% in the HSEP group and 26% in the SEP group) [26], which might explain the lack of substantial and statistically significant health benefits. 

To our knowledge, our study is the first cost-effectiveness analysis that directly compares the three exercise frameworks for patients with IC, providing valuable information for health-care planners and providers on appropriate interventions to prioritize. However, our study also has a few limitations that are important to consider. Mainly, the cost analysis captured IC-related in- and outpatient hospital costs, but not any potential primary care costs. It might be argued that exercise interventions have potential positive effects on other physical and mental conditions the patient might be suffering, which may lead to reductions in primary care use related to these conditions. However, given that we did not find any clinically meaningful or statistically significant differences in health outcomes, any such effects may be limited. Further, considering the health-care payer perspective in our analysis, we did not consider broader societal consequences such as potential effects on production loss or time use. However, considering the age profile of the patients, we believe that it is not likely that this would have caused any substantial differences to our results. Additionally, the HRQoL data suffered from missing data at different follow-up points in the study—a total of 56 missing HRQoL scores—that may have impacted the QALY outcomes. To account for this limitation, we used multiple imputation analyses as a robustness check, which led to very similar results as the complete case analysis. 

## 5. Conclusions

The results from this cost-effectiveness analysis showed that SEP combined with WA is more than twice as expensive as HSEP combined with WA and WA alone for patients with IC without providing any better health outcomes. The results also showed that in a choice between HSEP and WA, there is a 64 percent likelihood that HSEP is cost-effective at the informal Swedish health policy cost-effectiveness threshold. From a cost-effectiveness perspective, the results in this study therefore suggest that HSEP, but not SEP, might be a relevant intervention alternative over WA alone in this patient population.

## Figures and Tables

**Table 1:** Baseline demographics and risk factor profiles by intervention groups.

Number of Patients (%)	WA (n = 51)	HSEP (n = 48)	SEP (n = 50)
Mean age (SD)	72.5 (8.4)	71.6 (6.8)	72.3 (7.5)
Men:Women	31:20 (61:39)	28:20 (58:42)	29:21 (58:42)
Smoking habits			
Smoker	15 (29)	11 (23)	15 (30)
Before	30 (59)	34 (71)	31 (62)
Never	6 (12)	3 (6)	4 (8)
Comorbidity			
Diabetes Mellitus	15 (30)	16 (33)	14 (28)
Heart disease ^a^	16 (31)	12 (25)	16 (32)
COPD	5 (10)	7 (15)	7 (14)
Disease severity status (Rutherford classification)			
Mild	9 (18)	7 (15)	8 (16)
Moderate	34 (67)	28 (58)	32 (64)
Severe	8 (15)	13 (27)	10 (20)
Physically active before trial (30 min 3×/week)	17 (33)	14 (30)	14 (28)

^a^ Diagnosis of chronic heart failure, stable angina pectoris, or previous myocardial infarction. WA = Walk advice, HSEP = Home-based structured exercise program, SEP = Supervised exercise program.

**Table 2:** Mean HRQoL scores, QALYs, and cost per patient.

	WA (n = 51)	HSEP (n = 48)	SEP (n = 50)	*p*-Value
HRQoL scores, Mean (SD)				
Baseline (n = 131)	0.70 (0.13)	0.70 (0.12)	0.68 (0.11)	0.84
At 3 months (n = 122)	0.73 (0.13)	0.74 (0.11)	0.69 (0.13)	0.17
At 6 months (n = 117)	0.71 (0.12)	0.72 (0.12)	0.72 (0.14)	0.88
At 12 months (n = 122)	0.73 (0.12)	0.73 (0.13)	0.74 (0.12)	0.94
QALYs, Mean (95% CI)				
QALYs Complete case (n = 93)	0.72 (0.69–0.75)	0.74 (0.71–0.77)	0.73 (0.70–0.76)	0.77
QALYs Multiple imputation (n = 149)	0.72 (0.69–0.75)	0.72 (0.69–0.75)	0.71 (0.68–0.74)	0.86
Costs, Mean (95% CI)				
Outpatient costs	EUR 1291 (EUR 1111–EUR 1471)	EUR 1327 (EUR 1162–EUR 1491)	EUR 3410 (EUR 2946–EUR 3872)	<0.01
Inpatient costs	EUR 490 (EUR 0–EUR 1473)	EUR 493 (EUR 0–EUR 1012)	EUR 1209 (EUR 0–EUR 2423)	0.47
Total costs	1781 (EUR 717–EUR 2845)	EUR 1820 (EUR 1203–EUR 2438)	EUR 4619 (EUR 3227–EUR 6010)	<0.01

Abbreviations: HRQoL = Health-Related Quality-of-Life, QALYs = Quality-Adjusted Life-Years, SD= Standard Deviation, CI = Confidence Interval, WA = Walk advice, HSEP = Home-based structured exercise program, SEP = Supervised exercise program.

**Table 3:** Cost-effectiveness analysis based on complete case analysis for HSEP vs. WA, SEP vs. WA, and HSEP versus WA.

Comparison	Incremental Cost (95% CI)	Incremental QALYs (95% CI) *	Cost-Effectiveness Ratio
HSEP vs. WA	EUR 39.57 (−1194.83–1273.97)	0.01 (−0.04–0.08)	EUR 3749 per QALY
SEP vs. WA	EUR 2837.94 (1112.64–4563.25)	0.01 (−0.03–0.05)	EUR 278,981 per QALY
SEP vs. HSEP	EUR 2798.37 (1273.69–4323.05)	−0.0001 (−0.03–0.03)	HSEP dominates **

* Based on regression analysis controlling for baseline HRQoL score. ** HSEP has lower costs and better health outcomes (although the latter is not statistically significant). Abbreviations: CI = Confidence Interval, WA = Walk advice, HSEP = Home-based structured exercise program, SEP = Supervised exercise program.

## Data Availability

The data underlying this article cannot be shared publicly due to containing personal health information as determined by Swedish legislation.

## Supplementary Materials

The following supporting information can be downloaded at: https://www.mdpi.com/article/10.3390/jcm12165277/s1, Table S1: Outpatient cost categories and mean number of outpatient contacts per patient per intervention group with 95% confidence intervals. Table S2: Cost-effectiveness analysis based on multiple imputation analysis for HSEP vs. WA, SEP vs. WA, and HSEP versus WA.
