# Supplementary material for: Cost-Effectiveness of Exercise Therapy in Patients with Intermittent Claudication—A Comparison of Supervised Exercise, Home-Based Structured Exercise, and Walk Advice from the SUNFIT Trial
Source: J Clin Med. 2023 Aug 14;12(16):5277. doi: 10.3390/jcm12165277 (PMC10455939; doi:10.3390/jcm12165277)
Supplement: Supplementary file 1 [file jcm-12-05277-s001.zip › jcm-2494250-supplementary.pdf]

SUPPLEMENTARY MATERIAL

Table S1. Outpatient cost categories and mean number of outpatient contacts per patient per intervention group with 95% confidence intervals.

**Resource use** (average unit price)	**WA**		**HSEP**		**SEP**	
	Mean contacts	(95% CI)	Mean contacts	(95% CI)	Mean contacts	(95% CI)
Doctor contact (€ 328)	3.1	(2.6 – 3.7)	2.8	(2.2 – 3.2)	2.8	(2.4 – 3.3)
Nurse contact (€ 91)	0.4	(0.2 – 0.6)	0.3	(0.2 – 0.5)	0.8	(0.5 – 1.1)
Physiotherapist contact (€ 64)	3.7	(3.3 – 4.0)	6.2	(5.7 – 6.7)	37.7	(30.8 – 44.6)

Table S2. Cost-effectiveness analysis based on multiple imputation analysis for HSEP vs. WA, SEP vs. WA, and HSEP versus WA.

Comparison	Incremental cost (95% CI)	Incremental QALYs (95% CI)*	Cost-effectiveness ratio
HSEP vs. WA	€39.57<br>(-1,194.83–1,273.97)	0.01<br>(-0.04–0.06)	€7,960 per QALY
SEP vs. WA	€2,837.94<br>(1,112.64–4,563.25)	0.001<br>(-0.03–0.03)	€1,964,380 per QALY
SEP vs. HSEP	€2,798.37<br>(1,273.69–4,323.05)	-0.0001<br>(-0.03–0.03)	HSEP dominates**

* Based on regression analysis controlling for baseline HRQoL score. ** HSEP has lower costs and better health outcomes (although the latter is not statistically significant).
